# Supplementary material for: Combination Therapy after TACE for Hepatocellular Carcinoma with Macroscopic Vascular Invasion: Stereotactic Body Radiotherapy versus Sorafenib
Source: Cancers (Basel). 2018 Dec 14;10(12):516. doi: 10.3390/cancers10120516 (PMC6315557; doi:10.3390/cancers10120516)
Supplement: Supplementary file 1 [file cancers-10-00516-s001.pdf]

Supplementary material

Combination Therapy after TACE for Hepatocellular Carcinoma with Macroscopic Vascular Invasion: Stereotactic Body Radiotherapy versus Sorafenib

Lujun Shen, Mian Xi, Lei Zhao, Xuhui Zhang, Xiuchen Wang, Zhimei Huang, Qifeng Chen, Tianqi Zhang, Jingxian Shen, Mengzhong Liu and Jinhua Huang

Table S1. Number of repeats of TACE in each treatment group.

Number of Repeats	TACE-SBRT (*n*, %)		TACE-Sorafenib (*n*, %)
	Before SBRT	After SBRT	
0	0 (0.0)	0 (0.0)	0 (0.0)
1	13 (50.0)	1 (3.8)	21 (43.8)
2	13 (50.0)	2 (7.7)	14 (29.2)
3	0 (0.0)	1 (3.8)	5 (10.4)
4	0 (0.0)	0 (0.0)	4 (8.3)
5	0 (0.0)	0 (0.0)	2 (4.2)
6	0 (0.0)	0 (0.0)	2 (4.2)

© 2018 by the authors. Licensee MDPI, Basel, Switzerland. This article is an open access article distributed under the terms and conditions of the Creative Commons Attribution (CC BY) license (http://creativecommons.org/licenses/by/4.0/).
